# Flavor and Well‐Being: A Comprehensive Review of Food Choices, Nutrition, and Health Interactions

**DOI:** 10.1002/fsn3.70276

**Published:** 2025-05-16

**Authors:** Md. Sakhawot Hossain, Md Abdul Wazed, Sharmin Asha, Md Alomgir Hossen, Sk. Nur Muhammad Fime, Shamiha Tabassum Teeya, Lubna Yeasmin Jenny, Diptho Dash, Islam Md Shimul

**Affiliations:** ^1^ Department of Nutrition and Food Technology Jashore University of Science and Technology Jashore Bangladesh; ^2^ School of Nutrition and Public Health, College of Health Oregon State University Corvallis Oregon USA; ^3^ College of Food Science Sichuan Agricultural University Yaan China

**Keywords:** dietary preferences, eating behavior, flavor, nutrition, public health, taste

## Abstract

Human beings are naturally drawn to food flavors and pleasant aromas, which not only guide food choices but also contribute to health by promoting the intake of nutritious foods, aiding digestion, and enhancing emotional well‐being. This review explores the complex relationship between flavor, nutrition, and health, highlighting that flavor perception can be affected by genetic susceptibility, age, culture, gender, and early life experiences. They influence emotional and physiological responses through brain mechanisms, directly affecting food selection and health outcomes. The use of natural flavors enhances the taste of food and encourages healthier food choices. In contrast, the widespread use of artificial flavors, while often boosting food sales, often leads to the overconsumption of less nutritious products, thereby increasing potential health risks. There is a growing trend among health‐conscious consumers that shows a preference for natural and organic flavors, despite challenges such as low bioavailability and limited evidence of their effectiveness. However, advancements in food processing technologies such as microencapsulation and novel extraction methods offer promising tools to improve flavor stability and sensory acceptance, making healthier products more appealing and widely acceptable. In addition, the use of flavor in a strategic manner is most relevant in food reformulation, dietary interventions, and nutrition education, where it can influence consumers to make more health‐conscious and sustainable food choices. Subsequent research needs to focus on human trials to optimize flavor delivery techniques and dosages, along with the role of genetic traits and environmental influences on customized flavor perception. Governments across the world need to impose stricter regulations on synthetic additives to ensure safety and safeguard consumer health.

## Graphical abstract

This review presents an overview of the connection between flavor perception and human health, emphasizing the roles of natural and artificial flavors in influencing dietary behavior and nutritional outcomes. It synthesizes current evidence on how flavor influences food preferences, consumption patterns, and associated physiological responses. The article highlights the potential of natural flavors to support healthier eating habits, while also addressing concerns related to the overuse of artificial flavorings. Through sensory, neurobiological, and behavioral perspectives, the review illustrates the multifaceted impact of flavor on nutrition and well‐being.

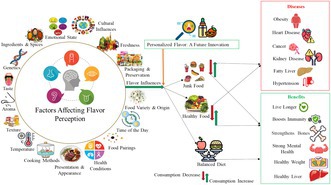

## 1 Introduction

Flavor has a significant impact on both physical health and mental wellness, both of which are crucial to overall well‐being. It affects dietary choices, preferences, palatability, and daily consumption decisions both before and after ingestion. Flavor is a multi‐sensory experience composed of taste, smell, and texture. Taste involves the perception of five fundamental sensations detected by taste receptor cells: umami, sourness, sweetness, bitterness, and salty (Tepper and Barbarossa 2020). Also, through the perception of odors, our sense of smell from the olfactory system regulates it to detect and identify different odors. Flavor perception depends on this sensation since it combines both aspects of taste with aromas that enhance our overall eating experience (Lee et al. 2024). Besides, texture refers to the functional and sensory properties of foods detected through touch, hearing, vision, and kinesthetics, providing information about their structural composition (Gallego et al. 2022). Flavor perception occurs when signals from our taste buds, nose, and mouth are processed together in the brain, particularly in areas such as the gustatory cortex and orbitofrontal cortex, resulting in the complete flavor experience. However, the molecular mechanisms behind odor‐taste interactions that influence flavor perception remain unclear (Chen et al. 2023). Food taste is a major determinant of eating habits, health, and quality of life. It has been observed that palatability enhanced by flavor, texture, and aroma improves food enjoyment, leading individuals to consume more healthy foods (Forde 2024). Recent sensory food research demonstrates that the intensity, variety, and balance of flavors play a key role in our food preferences, satiety, and portion sizes. In particular, enhancing umami makes vegetables and plant proteins more enjoyable to eat. By combining free amino acids and nucleotides, cooking methods can enhance umami, making healthy foods more appealing and facilitating the transition to a more sustainable diet (Schmidt and Mouritsen 2022). In contrast, unpleasant tastes can lead to food aversions and anorexia, resulting in low intake of certain nutrients and potentially negative health implications (Labrie et al. 2020). Moreover, the perception of flavor encompasses the cultural and social dimensions of eating, establishing food traditions and customs that influence dietary choices (Ali et al. 2022; Rodríguez‐Ramírez et al. 2022). The interaction between flavor and appetite regulation underscores its importance in promoting healthy eating behaviors, as flavors can either stimulate or discourage food intake based on individual preferences and experiences (Labrie et al. 2020; Roy and Kumar 2019). Unhealthy diets are a major factor in the global rise of non‐communicable diseases (Forray and Borzan 2024), making it crucial to understand how flavor impacts food choices. Behavioral nutrition science is gaining interest in harnessing flavor in order to achieve healthier consumption, particularly through minimization of salt, sugar, and fat without compromising taste. Methods like emulsion systems are being developed in order to limit these ingredients but retain sensory value, addressing the consumer's preference for healthier but flavorful products (Huang et al. 2024). In addition, studies have shown that healthier‐tasting foods are perceived as fewer in calories and of higher nutrition, and this has a positive effect on purchasing behavior (Cai et al. 2023). Therefore, flavor is not merely a chemical sense but a crucial component affecting individual health and broader population eating patterns.

As a vital aspect of nutrition, flavor shapes eating decisions and overall health by influencing nutritional intake as well as metabolic and mental well‐being. Due to this interconnection between flavor and health, understanding flavor's role is significant for promoting health and preventing or mitigating certain non‐communicable diseases. Previous studies have shown that flavor perception influences dietary choices and nutrient intake, which, in turn, affects overall diet quality. Individuals with greater flavor sensitivity are likely to eat more diverse diets, which could improve diet quality. But further evidence is needed to establish a clear causal relationship (Ozturk and Dikmen 2022). Increasing the flavor of nutritious foods may similarly enhance their intake, potentially contributing to weight management and reducing the risk of non‐communicable diseases like obesity, diabetes, and cardiovascular diseases. However, there has been limited research on the specific role of flavor in health outcomes. Therefore, this review synthesizes current evidence on how flavor influences dietary behavior and health outcomes, aiming to inform strategies that promote healthier eating patterns and improved nutritional status. By examining both natural and artificial flavors, the review aims to highlight their impact on palatability and dietary patterns. It also identifies key research gaps and offers insights to inform nutritional strategies and public health interventions that support healthier and more sustainable eating habits.

## 2 Flavor and Nutritional Well‐Being

### 2.1 The Psychological and Physiological Impact of Flavor on Eating Behavior

Flavor perception in humans plays a significant role in both psychological and physiological processes. Psychologically, flavor influences emotions and desire, while physiologically, it stimulates appetite and digestion, impacting nutrient absorption and overall health. The complex relationship among taste, odor, and physiological signals that regulate food intake highlights flavor's role in general health (Gallego et al. 2022; Tepper and Barbarossa 2020). According to Mastinu et al. (2023), taste and smell stimuli induce physiological responses that, in turn, influence the emotional responses to foods. These emotional responses can range from pleasure to disgust, directly influencing food preferences and eating behaviors. Positive emotions tend to increase the desire to eat a certain food, while negative emotions may lead to its avoidance (Mastinu et al. 2023). For example, our bodies respond to changes in flavor with physiological signals like changes in heart rate and sweating, which indicate whether we like or dislike a food. These responses help explain why certain flavors are perceived as pleasant and how they influence our preference for the consumption of a certain dish (Lagast et al. 2020). These sensory inputs are integrated into the brain, particularly in the taste cortex and orbitofrontal cortex, ultimately modulating flavor perception and influencing food choices and eating behaviors (Rolls 2022). Flavor, at the neural level, affects both energy balance and reward perceptions. For example, glucose, the component that gives the sweet taste, activates neurons in the hypothalamic and ventral tegmental area more strongly than ethyl‐butyrate, a compound that gives a fruity flavor. This activation modulates our feelings of hunger, energy regulation, and pleasure associated with eating (van Opstal et al. 2019). Additionally, ectopic olfactory receptors activated by flavor compounds have been linked to the regulation of adipogenesis, myogenesis, and hepatic lipid accumulation (Tong et al. 2021). Flavor aldehydes found in e‐liquids (liquid used in e‐cigarettes) can undergo chemical modification, producing neurotoxic and cytotoxic aldehyde acetals—known activators of sensory irritant receptors and mitochondrial toxicants (Jordt et al. 2020). These compounds affect neurobiology by increasing nicotinic acetylcholine receptors, enhancing dopamine neuron activity, and releasing dopamine in reward‐related brain areas, thus promoting vaping behaviors in a sex‐dependent manner (Cooper et al. 2023). Moreover, flavor can also lead to taste aversion through conditioning, where a negative event, such as nausea after eating a certain flavor, teaches the individual to avoid that flavor. Studies have shown that rats will avoid certain flavors even when the pleasant taste of a particular food is associated with discomfort (López‐Espinoza et al. 2022). This phenomenon illustrates the complex psychological and physiological responses to flavor, as summarized in Table 1

**TABLE 1:** Psychological and physiological responses to flavor.

Flavor Type	Psychological response	Physiological response	Health implications	References
Sweet	Pleasure, comfort	Stimulates salivation and triggers insulin secretion	Often linked to overconsumption, weight gain, and a higher risk of metabolic disorders such as diabetes	(Almiron‐Roig et al. 2023)
Bitter	Aversion, alertness	Suppresses appetite and activates liver detoxification enzymes	Supports detoxification processes, though aversion can reduce the intake of nutrient‐rich bitter foods	(Aronica et al. 2022)
Umami	Satisfaction, fullness	Facilitates protein digestion and promotes salivary flow	Enhances satiety and promotes adequate protein intake, contributing to appetite regulation and weight control	(Yeomans 2024)
Salty	Satisfaction, thirst	Promotes fluid retention and elevates blood pressure	Excessive intake increases the risk of hypertension, cardiovascular complications, and kidney dysfunction	(Modou et al. 2024)
Sour	Alertness, curiosity	Triggers salivation and stimulates digestive enzyme release	Stimulates appetite and digestion; excessive exposure can damage dental health by wearing away the hard, protective surface of the teeth	(Bozorgi et al. 2020; Chauhan et al. 2022)

**FIGURE 1:**
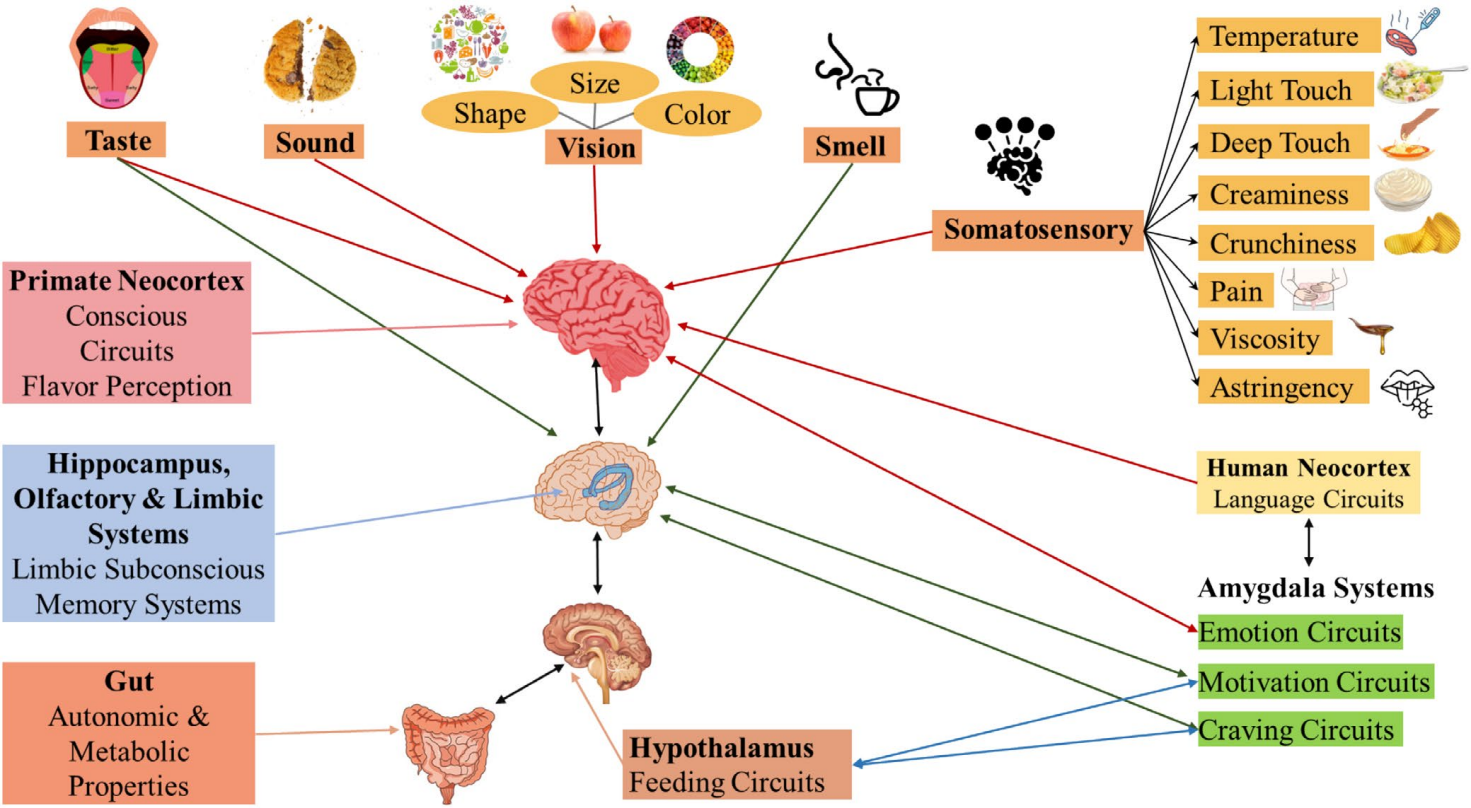
Schematic of human brain flavor systems regulating food intake.

Flavor plays a crucial role in food choices and eating habits by influencing preferences and satisfaction. It influences not only which foods we select but also how much we enjoy them. Taste is a key factor in regulating consumer food intake and influencing repeat consumption (Zhu 2024). This has significant implications for health and well‐being, as it can enhance the palatability of nutrient‐dense foods low in sugar, salt, fat, and alcohol, and eliminate off‐flavors in plant‐based protein products (Salta and Xiaofen 2024). Moreover, oral health and taste are closely related, as dental issues or treatments can affect the perception of taste and this can, in turn, affect food selection and intake (Ellender and Moynihan 2021). Early exposure to a variety of flavors can shape a child's long‐term taste preferences. Oral health as well as early experience with flavors play crucial roles in the formation of the sense of taste and food eating. Similar to how dental issues can affect taste perception and eating habits, early exposure to a wide range of flavors can have a lasting impact on an individual's taste preferences and dietary behaviors (Nicklaus and Tournier 2022). Individual differences in taste perception and the pleasure people get from eating can contribute to obesity, as taste plays a key role in controlling hunger, food cravings, and how much we eat (Tepper and Barbarossa 2020). A pleasant flavor in fruits and vegetables can increase nutrient intake, while an unpleasant flavor can reduce satisfaction, consumption, and overall well‐being (Labrie et al. 2020). Taste represents one of the most important human functions, helping us perceive the external world, select nutrients, avoid harm, and sustain life. Different tastes signal specific nutrient profiles: sweetness signals carbohydrates as energy sources, while salty taste suggests the presence of sodium and other vital salts, which regulate fluid concentration in cells and balance the osmotic pressure, promoting nutrient absorption. Umami reflects the amino acid content in food (Mutchler et al. 2021). Bitter and sour taste often identify potentially toxic substances or spoiled food, forming an innate mechanism of nutrient intake and protection (Liszt et al. 2022). Recent research has focused on investigating the brain's mechanism for processing taste and odor signals, which can affect dietary habits and food choices (Lee et al. 2024; Tepper and Barbarossa 2020). Different senses work together to help us perceive flavor. The hippocampus, which is responsible for memory, and the amygdala, which handles emotions, help control how much we eat. The neocortex, found in humans and primates, is responsible for our conscious awareness of taste. The hippocampus and hypothalamus also help regulate eating automatically, without us thinking about it (Rolls 2022; Tepper and Barbarossa 2020). This complex interaction is illustrated in Figure 1, which shows the human brain's flavor systems evaluating and regulating food intake.

### 2.2 The Influence of Flavor on Dietary Acceptance

Flavor is fundamental in promoting the optimal eating patterns, as it orients individuals toward healthy food options such as fruits, vegetables, and whole grains that are consistent with good dietary health. In contrast, flavor can also lead to overconsumption of less healthy, highly processed foods that are often engineered to be especially tasty. Studies have shown that yogurt with lighter colors, a smooth and creamy texture, a sweet taste, and flavors like coconut or vanilla can increase customer satisfaction and emotional responses (Cardello et al. 2024). Different flavors, therefore, may appear with specific emotional exaltations among men and women, which can guide food preference and consumption. For example, it is found that the flavor ‘chocolate’ generates a higher emotional response for men than for women (De Pelsmaeker et al. 2022), potentially enhance their desire for certain foods and affecting their dietary choices. The natural sweetness of fruits can reduce the intake of high‐calorie dessert (Mattes et al. 2023). Improving the flavor of food also presents a promising way for increasing meal acceptance. One study that added garlic and salt to enhance the taste of food found a significant increase in patient preference compared to the control group, suggesting that flavor enhancement could be an effective way to adjust meals to meet the sensory expectations of patients during treatment (Drareni et al. 2023a). Additionally, food flavor with salt and garlic significantly increased the liking of eggplant cream among cancer patients undergoing chemotherapy (Drareni et al. 2023b). This suggests that improving food flavor could enhance food acceptance and potentially help reduce the risk of malnutrition. Flavor has been a key factor in driving dietary choices and achieving positive nutritional outcomes across various populations. Successful interventions have shown that flavor modification can lead to healthier eating behaviors and good health outcomes. One pediatric nutrition intervention involved flavor enhancement, such as adding sweetness from fruit essences to vegetables and creamy textures to high‐fiber foods, to increase acceptance and nutrient intake among children (Lavelle 2023). Another study found that flavor adjustments increased the appeal of low‐calorie foods, aiding in weight management and adherence to dietary goals in adults (Lawler et al. 2024). Additionally, research with elderly participants showed that flavor enhancement at mealtimes improved food intake and dietary satisfaction, addressing age‐related declines in taste and smell, which supports better nutritional health (Chaffee and Ross 2023).

In summary, flavor influences emotional and physiological reactions, food preferences, and consumption through complex brain mechanisms and early experiences. These interactions have significant public health and nutrition consequences, especially with unhealthy diets continuing to propel the global non‐communicable disease burden. Utilizing flavor in food reformulation, dietary intervention, and nutrition education is an engaging strategy to support healthier and more sustainable eating patterns. Subsequent studies must further explore the manner in which particular flavor modification can facilitate a population shift to more nutrient‐dense, plant‐based, and lower‐sodium diets without compromise to taste.

## 3 Flavor Compounds and Their Health Implications

### 3.1 Health Benefits of Natural Flavors

The Food and Drug Administration (FDA) defines natural flavors as substances derived from natural sources, such as plant materials or animal products, through physical, enzymatic, or microbiological processes that correspond to those naturally occurring in nature (Queiroz et al. 2023). Common examples of natural flavors include the citrusy zest of lemon (Badiche‐El Hilali et al. 2024), the aromatic warmth of vanilla (Dibwe et al. 2023), the refreshing mintiness of peppermint (Kazemi et al. 2023), and the rich taste of cocoa (Tušek et al. 2024). These natural flavors, typically extracted as alkaloids and organic chemicals from plants, are considered healthier and often possess therapeutic properties (Singh and Sudha 2024). For example, vanillin from vanilla beans, commonly used in sweet foods, has antioxidant and anti‐inflammatory benefits (Dibwe et al. 2023). Similarly, ethyl butanoate, found in plant‐based and fermented foods like cheese and wine, provides a fruity flavor (Xu et al. 2020). Natural and synthetic sweeteners mainly provide sweetness with little to no calories. High‐potency sweeteners, which are much sweeter than sucrose, and bulk sweeteners, which offer the same or less sweetness as sucrose, are both commonly used. Natural sweeteners include sugar, sugar alcohols, steviol glycosides, and glycyrrhizin (Umar et al. 2023). Although fructose and glucose are common in natural sweeteners, they are used less frequently in the food industry compared to sucrose. Synthetic sweeteners like saccharin, aspartame, and sucralose are popular due to their high sweetness levels (Silva et al. 2023). However, natural alternatives such as polyols and D‐tagatose are gaining importance in the food industry because of their health benefits, clean labeling, and environmentally friendly synthesis (Castro‐Muñoz et al. 2022). Tagatose, which is an isomer of fructose, is recognized for its function as a flavor enhancer and a prebiotic, contributing positively to intestinal health and assisting in the regulation of blood sugar levels. Nevertheless, in spite of these advantages, its application remains limited in products such as cereals and ice cream due to various considerations, including cost and restricted availability (Wang et al. 2022). Flavonoids, carotenoids, and polyphenols are examples of bioactive compounds found in whole foods that not only contribute to natural flavors but also have antioxidant and anti‐inflammatory properties, supporting health by managing and preventing chronic diseases (Chandra 2022; Hossain et al. 2025). In addition to these benefits, these compounds promote well‐being by regulating hormones, supporting the immune system, and helping prevent diseases such as cancer and cardiometabolic disorders (Chandra 2022). Furthermore, many natural flavors, particularly the bitterness in foods and beverages, promote better digestion and gut health by increasing blood flow to the digestive tract and slowing gastric emptying rate (McMullen 2022). Moreover, glycyrrhizin, derived from licorice root, is not only a high‐potency sweetener but also exhibits potential health benefits such as anti‐inflammatory and antioxidant properties (Gromova et al. 2022). Similarly, cinnamaldehyde, a spice component found in cinnamon used in food and fragrances, has been documented to confer protective benefits against arthritis, sepsis, diabetes, and ulcerative colitis, and is effective in treating inflammation, bacterial infections, cancer, cardiovascular diseases, and kidney diseases (Guo et al. 2024). These nutritional benefits of flavor compounds, along with their roles in disease prevention and health promotion, are summarized in Table 2, which highlights specific compounds, their sources, and associated health benefits.

**TABLE 2:** Flavor compounds and their nutritional benefits.

Flavor compound	Food source	Nutritional benefits	References
Capsaicin	Chili peppers	Supports anti‐inflammatory response, acts as an antioxidant, and contributes to metabolic health	(W. Zhanget al., 2024)
Flavonoids	Berries, citrus fruits	Provide antioxidant protection, help regulate inflammation, and reducing chronic disease risk	(Ebrahimi et al. 2023)
Quercetin	Onions, apples	Enhances cardiovascular function, controls blood pressure, and reduces oxidative stress	(Aghababaei and Hadidi 2023)
Glucosinolates	Broccoli, Brussels sprouts	Support detoxification processes and strengthen antioxidant defenses	(Flockhart et al. 2023)
Anthocyanins	Blueberries, blackberries	Improve cognitive health, reduce inflammation, and protect against cellular damage	(Avula et al. 2023)
Curcumin	Turmeric	Regulates inflammation and oxidative stress, supporting joint, and brain health	(Shahrajabian and W. 2024)
Eugenol	Cloves, cinnamon	Exhibits antimicrobial properties, provides pain relief, and supports dental health	(Elbestawy et al. 2023)
Limonene	Citrus peel	Enhances digestion and strengthens immune response through antioxidant activity	(Chen, Hassan, et al. 2024)
Catechins	Green tea, dark chocolate	Contribute to cardiovascular health, weight regulation, and cellular protection	(Sasaki et al. 2022)
Menthol	Mint	Provides digestive comfort, antimicrobial effects, and respiratory relief	(Kazemi et al. 2023)
Allicin	Garlic	Boosts immune response, helps manage blood pressure, and defends against microbial infections	(Ravindra et al. 2023)
Theobromine	Cocoa	Enhances mood, promotes heart health, and functions as a mild stimulant	(Cañas et al. 2023)
Piperine	Black pepper	Improves nutrient absorption, digestive efficiency, and metabolic health	(Raghunath et al. 2024)
Thymol	Thyme	Supports oral health, exhibits antimicrobial activity, and enhances immune function	(Dong, Yu, et al. 2023)

However, despite the significant antioxidant and anti‐inflammatory activities of natural flavor compounds such as vanillin and cinnamaldehyde, their actual health benefits depend on factors such as bioavailability, dose–response, and stability within the food matrix. For instance, vanillin is rapidly metabolized and excreted so that its availability and therapeutic effectiveness are limited to the system (Olatunde et al. 2022). Similarly, the effectiveness of cinnamaldehyde in treating long‐term diseases like diabetes and inflammation depends on the dose, the food it is combined with, and how the body processes it (Khare et al. 2016). Although these compounds are being added to more functional foods, there are not many clear studies showing how much people should take or which form works best. To move forward, future research should include human trials to find the right dosage, understand how the body processes them, and check long‐term safety. It should also look into advanced methods like microencapsulation or nanoemulsions to improve how stable and effective they are in the body. In addition, knowing how different bioactive compounds work with each other and establishing clear guidelines for the food industry regarding the use of these compounds and claiming health benefits from them will make natural flavors a part of healthy, science‐based food (Witkamp 2022). Conducting human trials, establishing appropriate dosages, optimizing delivery methods like microencapsulation or nanoemulsions, and formulating specific guidelines for the food industry are all key steps toward converting scientific observations about natural flavor compounds into effective health benefits and applications in the functional food market.

### 3.2 Influence of Artificial Flavors and Additives on Health

Artificial flavoring uses synthetic compounds to replicate or enhance specific tastes and aromas in food without using natural ingredients, such as floral or anise notes (Khantyanissa and Ervina 2024). Common artificial flavorings include vanillin (synthetic vanilla), ethyl maltol (a caramel flavor), and benzaldehyde (almond flavor). These substances are widely used in the food industry to mimic or enhance flavors (Dong et al. 2023; Song et al. 2024; Zhao et al. 2024). While artificial flavors and additives are commonly used to improve taste and extend shelf life, there is growing concern about their potential health impacts (Cox et al. 2024; Fallon et al. 2024; Nunes et al. 2023). For instance, a study conducted in North Carolina, USA, revealed potential health risks associated with artificial food colors, particularly for children, and found that 43.2% of grocery store products marketed to children contain these additives, especially in candies, fruit‐flavored snacks, and drink mixes (Batada and Jacobson 2016). Some artificial flavors and additives have been associated with adverse health effects, such as allergic reactions, gastrointestinal problems, and probably even long‐term effects of hyperactivity or metabolic disorders (Liang 2023). However, it is important to note that some studies have failed to establish a clear causal relationship between artificial additives and these health outcomes, suggesting that the risks may vary depending on the individual and the amount of exposure (Warner 2024). One study found that long‐term intake of saccharin and cyclamate mixtures led to oxidative stress, impaired glycemic control, and an increased risk of atherosclerosis, as well as hepatic and renal dysfunction, in both healthy individuals and those with diabetes (Hasan et al. 2023). Higher consumption of artificial sweeteners was related to an increased risk of total cardiovascular and cerebrovascular diseases. Specifically, aspartame was linked to a higher risk of cerebrovascular events, while acesulfame potassium and sucralose were associated with an increased risk of coronary heart disease (Debras et al. 2022). Additionally, artificial sweeteners like aspartame and acesulfame‐K, commonly used in food and drinks worldwide, have been linked to a higher cancer risk (Debras et al. 2022). These findings highlight the ongoing debate in the scientific community about the long‐term safety of artificial additives, as the evidence remains inconclusive. Certain additives, such as artificial colorings and preservatives, are also potential carcinogens and endocrine disruptors (Liang 2023). However, some studies offer conflicting evidence regarding the health risks of artificial additives. For example, Savin et al. (2022) found that while some children may show mild reactions to food colorings, the vast majority consuming artificial additives showed no adverse effects, suggesting a threshold effect rather than a universal risk (Savin et al. 2022). This finding aligns with other research, which has suggested that artificial additives are generally safe when consumed within typical dietary levels (Warner 2024). After years of research, health advocacy groups petitioned the FDA to ban the use of seven commonly used artificial flavors that have been shown to cause cancer in lab animals (Britt Erickson 2018). This contrast between the health risks of artificial flavors and the benefits of natural flavors is detailed in Table 3, which provides a comparative analysis of their health implications.

**TABLE 3:** Comparative overview of the health benefits of natural flavors and risks of artificial flavors.

Flavor sources	Use in food products	Health benefits	Health risks	References
Natural				
Vanilla extract	Beverages, and pharmaceuticals	Potentially helps prevent hepatosteatosis and supports liver health	N/A	(Dibwe et al. 2023)
Lemon zest	Baked goods, marinades, desserts, and beverages	Rich in antioxidants, exhibits anti‐inflammatory effects, supporting immune function	N/A	(Badiche‐El Hilali et al. 2024)
Mint leaves	Teas, desserts, salads, sauces, and beverages	Aids digestion, has anti‐inflammatory and antimicrobial properties, may relieve symptoms of irritable bowel syndrome (IBS), freshens breath	N/A	(Best 2023)
Cinnamon	Baked goods, beverages, savory dishes, and desserts	Contains antioxidants, has anti‐inflammatory properties, may lower blood sugar levels, supports heart health	N/A	(Guo et al. 2024)
Ginger	Teas, baked goods, savory dishes, sauces, and beverages	Anti‐inflammatory, aids digestion, relieves nausea, contains antioxidants, supports immune health	N/A	(Shaukat et al. 2023)
Cocoa	Chocolates, baked goods, beverages, and desserts	Rich in antioxidants, improves heart health, enhances mood, has anti‐inflammatory properties	N/A	(Tušek et al. 2024)
Almond extract	Baked goods, desserts, beverages, and sauces	Supports heart health, provides antioxidant properties	N/A	(de Souza et al. 2020)
Orange peel	Baked goods, teas, sauces, marmalades, and beverages	Rich in Vitamin C, contains antioxidants, supports immune health, improves digestion	N/A	(Nagar and Rastogi 2022)
Peppermint	Teas, candies, gums, baked goods, and sauces	Aids digestion, relieves nausea, has antimicrobial properties, may reduce headaches and improve breath	N/A	(Kazemi et al. 2023)
Nutmeg	Baked goods, desserts, sauces, and beverages, spice and flavor enhancer, and instant drinks	Contains antioxidants, supports digestion, may improve cognitive function, has anti‐inflammatory properties and anti‐diabetes		(Kaur et al. 2024)
Artificial				
Vanillin (artificial vanilla)	Candies, baked goods, and processed foods	N/A	May contain chemicals linked to allergic reactions, potential carcinogens, and reduced nutritional benefits	(Zhao et al. 2024)
Ethyl vanillin	Candies, baked goods, processed foods, dessert, baked goods, and tobacco	Antioxidant, anti‐inflammatory, antibacterial, anticancer, and neuroprotective properties	Cytotoxic effects on osteoblasts during bone‐forming processes and induce energy pathway dysfunction and cellular stress responses in a renal model	(Cox et al. 2024; Yun et al. 2024)
Benzaldehyde (artificial almond)	Soft drinks, candies, baked goods, puddings, and meat	Promotes drug absorption by enhancing membrane permeability and reducing oxidative stress and supporting brain cell survival	Risks may include hepatotoxicity and hallucinations	(Song et al. 2024)
Isoamyl acetate (artificial banana)	Candies, baked goods, syrups, honey, butterscotch, artificial coffee, and beverages	N/A	Showed significant toxicity, cytotoxicity, and genotoxicity	(Barros Rocha et al. 2018; Yusoff Azudin et al. 2020)
Ethyl butyrate (artificial pineapple)	Candies, sodas, and processed foods	N/A	Exhibits acute toxicity in in vitro and in vivo tests, causing behavioral changes and disrupting intestinal motility in mice	(Nunes et al. 2023)
Methyl anthranilate (artificial grape)	Candies, beverages Baked goods, ice creams, and chewing gums	N/A	Allergic reactions, cytotoxicity and renal stress	(Fallon et al. 2024)
Diacetyl (artificial butter)	Popcorn, baked goods, and processed foods	N/A	Health threat of respiratory inflammation and obstruction. Poses health risks including severe lung disease even at low exposure levels. Genotoxic and liver toxic effects in mice	(Barros Rocha et al. 2018; Nunes et al. 2023; Salama et al. 2023)
Ethyl maltol	Cigarettes, confections, beverages, meat products, jelly, candies, and even savory items.	N/A	May induce allergic reactions in sensitive individuals, manifesting as skin irritation and respiratory symptoms	(Dong, Yu, et al. 2023; Durrani et al. 2021)
Ethyl acrylate	Beverages and confectionary	N/A	Tested for allergic reactions and hypersensitivity, which may cause skin irritation, respiratory issues, or gastrointestinal discomfort in sensitive individuals	(Singh and Sudha 2024)
Artificial mint flavor	Candies, chewing gums, and oral hygiene products	N/A	Allergic reactions, gastrointestinal issues, and possible long‐term health effects	(Sambu et al. 2022)
Benzophenone	Ice creams	N/A	Potential cancer‐causing agent, with long‐term exposure possibly increasing the risk of developing cancer	(Singh and Sudha 2024)

## 4 Cultural and Individual Differences in Flavor Perception

### 4.1 Cultural Determinants of Flavor Preferences and Dietary Patterns

Food choice is a very complex human behavior and is influenced by environmental, socio‐cultural, and biological factors. Food culture refers to the accumulated practices, traditions, and preferences of food that a certain community or society possesses. It encompasses not only what people eat but also how, when, and why they eat, reflecting wider social and cultural dynamics (Criss et al. 2020). Different cultural contexts foster different flavor profiles, determined by local ingredients, culinary traditions, and historical factors. For example, the spicy, bold flavors common in Mexican cuisine are contrasted by milder, more subtle flavors found in Scandinavian diets. These preferences for specific flavors are inherently connected to a culture's environment and available resources (Reddy and van Dam 2020). Cultural, racial, and ethnic identities are some of the critical social determinants that strongly influence dietary habits. These factors, along with geographical location, climate, and historical context, collectively influence food traditions and dietary choices. For example, a variety of spices and herbs form integral parts of most South Asian diets, while fermented products form an important part of East Asian cuisines (Surya 2024). Such is the cultural impact on foods that, besides dictating the choice of food intake, the dietary patterns also vary significantly among different regions. For instance, aboriginal people such as Aboriginal Australians innately consume bush foods with naturally bitter greens and wild game meat, thereby establishing a personal taste system from environmental adaptation (Jones and Clarke 2018). Similarly, West African diets commonly include fermented, bitter, and hot ingredients, such as ogiri and locust beans, that reflect ecological appropriateness and preservation needs (Karamba and Abdullahi 2022). Food preferences and appetite can also be influenced by sensory exposure, such as repeated exposure to certain food odors (Zhang et al. 2024). Cultural norms and practices shape these exposures over time, contributing to the development of taste preferences within specific communities. For example, veganism and vegetarianism are the changes in food consumption culturally presented in which economy showed the values that have changed related to health, ethics, and environmental sustainability. Diets are on demand all around the globe; from 23 countries, veganism is most on demand (Laureati et al. 2020), illustrating how global food culture is evolving in response to new societal trends. The concept of “local flavor,” or the local cuisine that reflects cultural heritage, promotes diversity and can express a community's history, values, and traditions (Putri and Wijoyo 2023). Cultural factors strongly influence the development of taste and food habits, with traditional practices, available ingredients, and cultural norms shaping unique tastes and food choices across different cultures (Zhang et al. 2023). Comparing different cultural diets delineates how regional cuisines are often centered around specific flavors and food groups, such as the use of spices in Indian cuisine or fresh ingredients in Mediterranean diets (Ghosh 2022). In contrast, in the USA, preferences for salty and sweet tastes drive the consumption of processed foods, snacks, and fast foods like burgers, fries, and sugary beverages, influenced by taste appeal, convenience, marketing, and availability (Grasso et al. 2023). The diverse flavors in Asian cuisine represent rich culinary traditions: East Asia frequently uses umami flavors like soy sauce and miso, while Southeast Asia balances sweet, salty, sour, and spicy tastes in dishes such as Thai curry and Vietnamese pho, which incorporate fresh herbs, spices, and fermentation techniques (Somnuk et al. 2023). The factors affecting food practices include access, environment, nutritional needs, and social or psychological influences. Access to ingredients shapes flavor profiles from region to region. The environment controls the type of spices or herbs used. Nutritional requirements determine flavor preferences, e.g., a craving for umami taste in protein‐containing foods. Further, cultural and social factors, coupled with psychological comfort food aspects, play a big role in how people perceive flavors. In Singapore, cross‐cultural food practices are common and influenced by health concerns, variety, taste, and convenience (Reddy and van Dam 2020). Traditional food practices, culinary customs, and dietary preferences rooted in cultural heritage offer valuable insights into the nutritional value of different foods (Hamed Kandil 2022). There is evidence that people's enjoyment of food odors (hedonics) and their familiarity with certain scents can change with the seasons, particularly in countries with distinct seasonal climates. This means our liking and perception of foods are subjected to the annual rhythm, whereby at particular times of the year, certain smells and flavors turn more appealing (Spence 2021). As shown in Table 4, cultural influences play a crucial role in shaping flavor preferences and dietary habits, which in turn have important health implications.

**TABLE 4:** Cultural determinants of flavor preferences, dietary habits, and related health outcomes.

Culture or country	Common flavor preferences	Typical dietary habits	Health outcomes	References
USA	Sweet, salty, and savory	Processed foods, high sugar intake, fast food, large portions, meat, dairy	High rates of obesity, diabetes, heart disease, risk of nutritional deficiencies with poor diet quality	(Chen, Ding, et al. 2023; Grasso et al. 2023)
Japan	Umami, salty, and sweet	Rice, fish, seaweed, fermented foods, minimal red meat	Low rates of heart disease, high life expectancy, risk of stomach cancer from high salt intake	(Tanaka et al. 2023; Tsugane 2021)
Bangladesh	Spicy, savory, and sweet	Rice, lentils, fish, vegetables, minimal meat, diverse spices	High rates of malnutrition, low obesity rates, risk of nutrient deficiencies, emerging non‐communicable diseases with dietary changes	(Ahmed et al. 2022; Ali et al. 2022; al Banna et al. 2022; Liza et al. 2024)
Nigeria	Spicy, savory, and starchy	High consumption of dietary salt, red meat, dietary fat, junk food, and alcohol. Yam, cassava, rice, beans, leafy greens, limited meat	High rates of malnutrition and infectious diseases, risk of hypertension with high salt intake, increasing obesity rates	(Batubo et al. 2023; Ecker et al. 2020)
Mexico	Spicy, savory, and sweet	Corn‐based dishes, beans, chili peppers, fresh vegetables, limited dairy	High rates of obesity and diabetes, nutrient‐rich traditional diet, risk of high sodium intake	(Rodríguez‐Ramírez et al. 2022)
Italy	Savory, sweet, and bitter	Pasta, olive oil, fresh vegetables, lean meats, cheese, moderate wine consumption	Mediterranean diet linked to low heart disease, risk of obesity increasing with Westernized diet	(Dominguez et al. 2023)
India	Spicy, aromatic, and sweet	Rice, lentils, vegetables, diverse spices, limited meat	High rates of diabetes, varied diet, risk of heart disease with increasing processed food consumption	(Sachdev and Misra 2023)
China	Umami, salty, and spicy	Rice, noodles, vegetables, soy products, seafood, minimal dairy	Increasing obesity and diabetes rates with Westernized diet, traditionally low heart disease rates	(Qin et al. 2021)
France	Savory, sweet, and rich	Bread, cheese, wine, fresh vegetables, lean meats, pastries	Low rates of heart disease (French Paradox), risk of obesity with increasing processed food consumption	(Beslay et al. 2020)
Thailand	Spicy, sweet, and sour	Rice, noodles, seafood, fresh herbs, coconut milk	Low obesity rates, risk of hypertension from high sodium intake	(Somnuk et al. 2023)
Greece	Savory, sweet, and bitter	Olive oil, vegetables, legumes, fish, moderate meat consumption	Mediterranean diet linked to low heart disease, high life expectancy, risk of obesity with increasing Westernized diet	(Benyaich 2017)
Brazil	Savory, sweet, and tropical	Rice, beans, fresh fruits, vegetables, meat, seafood	Increasing obesity and diabetes rates, risk of heart disease with high meat consumption	(Machado et al. 2022)

### 4.2 Individual Differences in Taste Sensitivity and Food Preference

Individual variation in taste sensitivity and preference is influenced by genetic and physiological factors. Genetic differences play a role in the perception of the basic tastes—sweet, salty, sour, and bitter (Piluso et al. 2024). Individual differences in taste sensitivity and preference are determined by age, gender, neurotype, and other determinants. Additionally, the concentration of taste buds itself has a very important effect on the differences (Melis et al. 2020). Evidence suggests that obese women tend to have a stronger preference for sweet‐fat foods, while obese men show a stronger preference for savory‐fat foods. Furthermore, taste sensitivity varies with age, and women's concern for health may lead them to make healthier choices for themselves and their families (Ullah et al. 2023). Research also reveals that children with neurodevelopmental disorders are particularly sensitive to taste and smell, and such sensory sensitivities are linked to food fussiness (Ide‐Okochi et al. 2024). On the other hand, personality characteristics, like neuroticism, can influence food choices, with neuroticism being a strong modulator of food preferences, particularly in obese individuals who show a higher preference for sweet tastes (Spinelli and Monteleone 2021). Consumer preferences for food texture, especially in European children, point to the influence of regional food habits on vegetable and legume intake. Food neophobia and intake of healthy food are influenced by whether a person is a hard‐liker or soft‐liker. Hard‐likers, who like strong flavors, tend to have lower food neophobia and consume healthier foods at a higher rate. On the other hand, soft‐likers, who prefer weaker flavors, may show more food neophobia and thus tend to consume fewer healthy foods (Laureati et al. 2020). Older adults generally have higher detection thresholds for taste and report weaker taste intensity compared to younger adults (Jeon et al. 2021).

### 4.3 Genetic, Age, and Gender Influences on Flavor Perception and Food Choice

**FIGURE 2:**
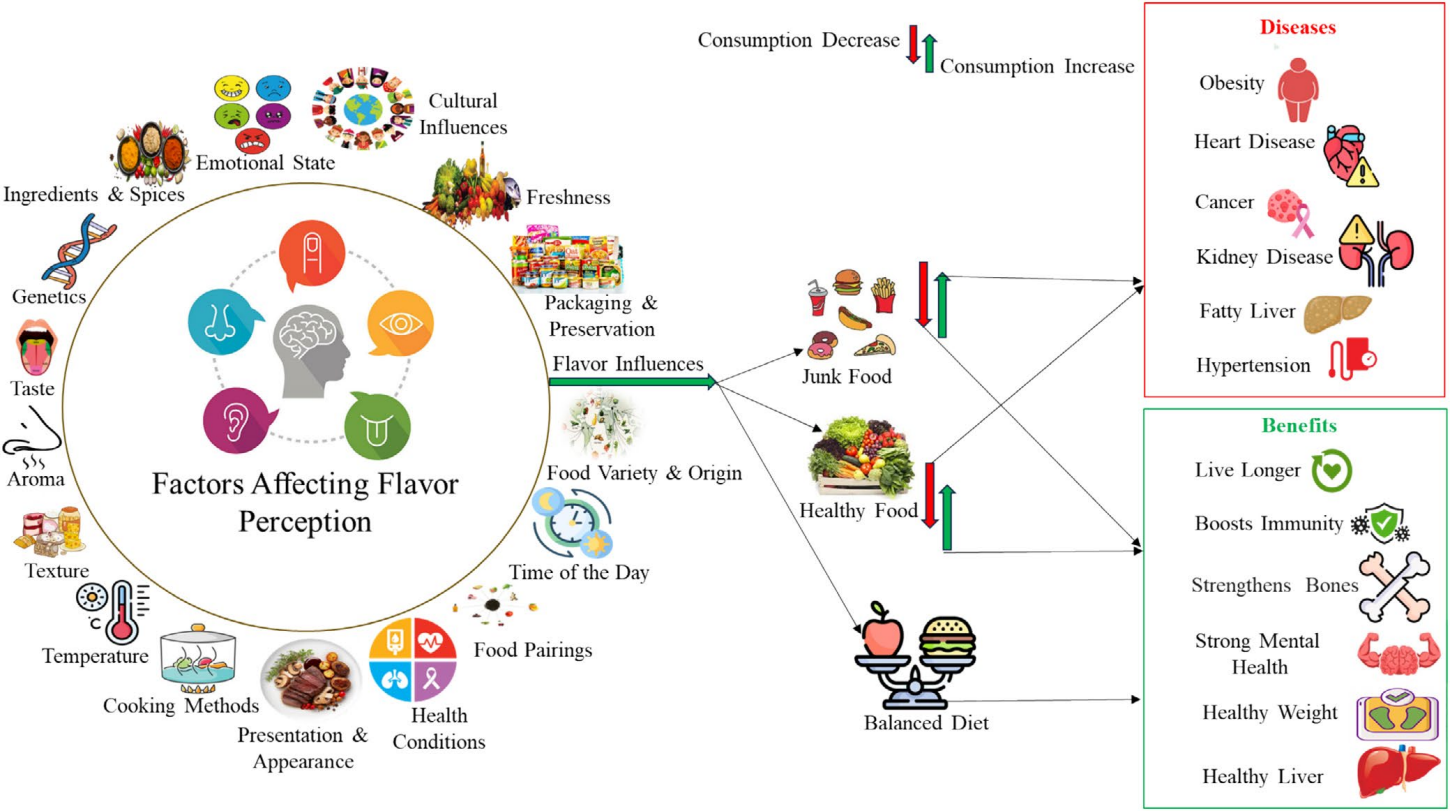
Interaction of flavor perception on dietary behavior and health outcomes.

There is clear evidence that consumers experience different sensory worlds and food preferences influenced by genetic differences (Chen et al. 2024). Genetic variations, especially in the gene TAS2R38, which codes for bitter taste receptors, are linked to differences in food acceptance, dietary patterns, and ultimately health disparities. In high‐altitude regions with limited fresh produce, such as parts of the Andes, populations adapt to bitter and umami‐rich fermented foods, potentially overriding innate genetic preferences due to necessity and tradition (Majumder and Bhattacharya 2024). These variations alter taste sensitivity and influence behaviors related to diet, alcohol intake, tobacco use, and disease susceptibility, thereby impacting food preference and consumption (Chenet al. 2024). The TAS1R2 gene is associated with sweet taste perception (Stevens et al. 2024), while the SCNN1B gene is linked to salty taste perception (Cecati et al. 2022). Specifically, the rs35874116 polymorphism in the TAS1R2 gene affects sweet taste preference, diet choices, and health outcomes (Stevens et al. 2024). Age‐related changes in taste cells, reduced salivary secretion, and less efficient chewing are key factors that may affect taste perception in aging individuals. Poor taste receptor cells due to poor oral health and declining olfactory function may be responsible for the taste loss in older adults (Brown et al. 2024). Interestingly, beyond age, gender also appears to influence taste perception; women have been found to perceive all taste intensities more strongly than men, although the difference is least pronounced for umami (Ullah et al. 2023). Figure 2 illustrates factors affecting flavor perception and their impact on dietary choices, highlighting the link between flavor and health risks and emphasizing its role in overall well‐being.

The evidence shows that flavor perception is influenced by a combination of genetics, age‐related changes, and cultural factors. Although genetic traits such as TAS2R38 and TAS1R2 play a role in determining basic taste sensitivity, environmental and cultural factors can also modify these responses. Understanding these factors is important for creating personalized dietary recommendations and improving our knowledge of how taste relates to nutrition and health.

## 5 Mechanisms Linking Flavor to Health Outcomes

### 5.1 Neural and Hormonal Pathways Connecting Flavor Perception to Health

Food undergoes complex mechanical, thermal, and chemical interactions during chewing, which influence the perception of texture and flavor (Oppen et al. 2023). Various parts of the brain participate in the process: the insular cortex processes taste information and integrates it with texture and temperature (Schiff et al. 2023); the orbitofrontal cortex represents taste reward value (Rolls 2023); the anterior cingulate cortex and amygdala evaluate food rewards (Rolls 2023); and the hypothalamus integrates gut peptide signals with neural information from the mouth and gastrointestinal (GI) tract (Li et al. 2023), influencing flavor preferences and feeding behavior. The humoral pathway involves gut peptide hormones such as cholecystokinin (CCK), pancreatic polypeptide (PP), peptide YY (PYY), oxyntomodulin (OXM), and glucagon‐like peptide‐1 (GLP‐1), which affect food intake by signaling the arcuate nucleus of the hypothalamus (Hong and Choi 2024). Taste has multifactorial effects on health by monitoring toxins through gastrointestinal receptors that oversee nutrient intake, homeostasis, and energy metabolism (Xie et al. 2023). Bitter taste receptors in the GI tract play significant roles in metabolic and immunological responses, microbiota composition, and overall health. They regulate glucose via GLP‐1 induction and are modulated by traditional medicines like steviosides. Genes like T2R4 and T2R14 are associated with glucose control and anorexigenic activity, and these receptors are also responsible for signaling the presence of toxins, helping the body avoid them (Morini 2024). The mechanism described above can be harnessed to develop new drug targets or include ligands of bitter taste receptors in medications or nutraceuticals for metabolic control and general health. Modulating motility and satiation by targeting gastrointestinal taste receptors could lead to treatment for diseases such as obesity and diabetes (Kato and Oshima 2023). G protein‐coupled taste receptors for sweet, umami, and bitter tastes in the gut play roles in nutrient sensing, hormone release, and microbiota composition. They are also involved in immune response and assist in the removal of noxious substances from the colon, thereby helping to treat disorders of the gastrointestinal tract (Feng et al. 2022).

### 5.2 The Gut‐Brain Axis: Flavor's Role in Appetite Regulation and Well‐Being

**FIGURE 3:**
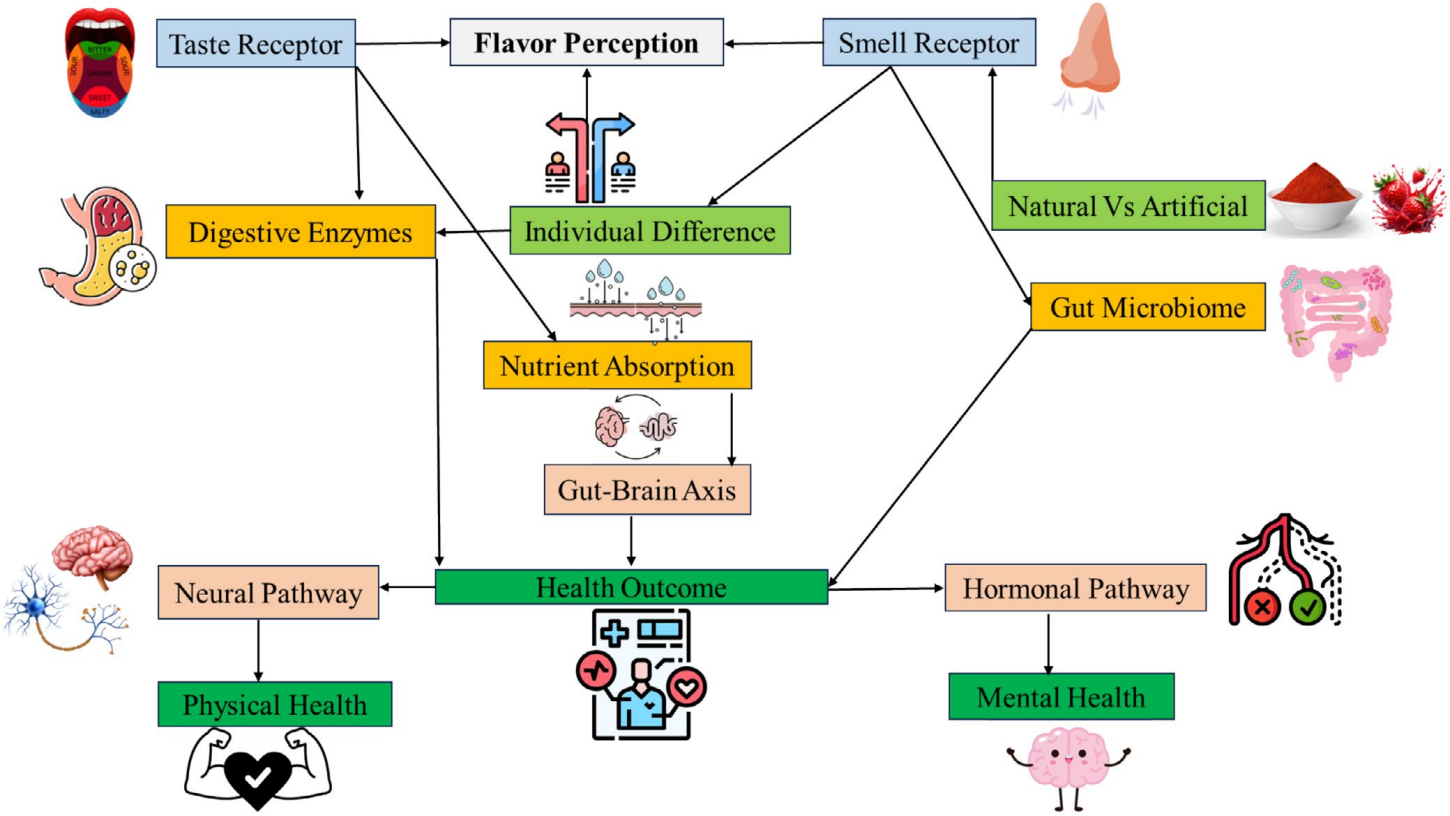
Sensory and behavioral links between flavor and health.

The Gut‐Brain Axis is a bidirectional communication network between the gut and the brain that influences digestion, appetite, and general well‐being. This includes neural, hormonal, and immune signals. Ghrelin, for example, triggers appetite through the hypothalamus and sends signals to the mesolimbic reward circuit, influencing addiction, impulsive behavior, and food reward (Engel et al. 2023). Flavor perception can impact gut microbiota and metabolic processes, ultimately modulating mood and mental health. The hypothalamus balances energy by inducing satiety through hormones like CCK, GLP‐1, PYY, insulin, and leptin. These hormones are released by enteroendocrine cells in the GI tract in response to food and help to control food intake by signaling the vagus nerve (Barakat et al. 2024). Similar to the hypothalamus, the gut microbiota affects the gut‐brain axis and central satiety metabolism through the release of cytokines (de Wouters d'Oplinter et al. 2022. The gut microbiota can modulate GLP‐1 secretion, influencing appetite and the reward system through short‐chain fatty acids (SCFAs) and other bacterial metabolites like propionate (Angelini et al. 2024). Obesity has been related to reduced sensitivity to sweet and fat taste, which may be influenced by oral and gut microbiota, although genetic and non‐genetic determinants of taste perception are still being investigated (López‐Dávalos et al. 2023). Figure 3 shows the flow from flavor perception (taste and smell receptors) to biological processes (digestive enzymes, nutrient absorption, gut microbiome), which then interact with the gut‐brain axis (neural pathways, hormonal responses). This interaction ultimately affects health outcomes, including physical and mental health.

### 5.3 Flavor's Influence on Emotional, Psychological, and Behavioral Health

Flavor has a significant role in emotional and psychological health by modulating mood, memory, and stress levels. The taste of food interacts with gut microbiota, taste receptors, and gut hormones to modulate food choice, nutrient absorption, and gut homeostasis (Xie et al. 2023). The intersection of nutrition and mental health focuses on understanding how diet influences cognitive function, emotions, and mental well‐being, as well as how mental states shape food choices (Firth et al. 2020). Changes in taste perception can lead to changes in food choice and intake, hence affecting general eating behavior. Flavor experiences can have profound effects on psychological health. For instance, sweet flavors are often associated with comfort and reduced stress, while bitter tastes might signal caution or distaste (Davis and Running 2023). Flavor‐related nostalgia is linked to positive emotions and mood enhancement, contributing to overall well‐being (Simpson et al. 2024). Certain tastes, particularly those in spicy foods, can stimulate the release of endorphins, creating a feeling of euphoria (Rhyu et al. 2021). Flavors impact mood by evoking emotions associated with food, such as happiness, interest, joy, and tenderness (Mastinu et al. 2023). Studies have shown that flavors also affect psychological health by influencing emotional eating and food choices (Tuck et al. 2023). Emotional eating and depressive symptoms are positively correlated and can predict higher consumption of sweet and non‐sweet energy‐dense foods, while depressive symptoms are associated with lower intake of vegetables and fruits (Braden et al. 2023). Additionally, some foods contain unique components that can affect psychological health. For example, lemon essential oil components like limonene and citral have been shown to reduce both physical and psychological stress by inhibiting the elevation of stress hormones and brain neurotransmitters (Asikin et al. 2022). The current evidence highlights the complex role of flavor in health, mediated by neurobiological, hormonal, and microbial pathways. Flavor not only influences food preferences and intake but also interacts with gut‐brain signaling and emotional health. Critically, understanding these mechanisms can inform targeted interventions for improving both metabolic and psychological outcomes, suggesting flavor as a promising focus for future nutrition and health research.

## 6 Current Challenges and Research Gaps

### 6.1 Issues Related to the Dominance of Processed Foods

Processed foods with artificial flavorings are considered an important health and nutritional concern worldwide. A recent study revealed that nearly half of the commercially produced complementary food products in Southeast Asia are highly processed and often contain high levels of sugar, sodium, and artificial additives like flavors, colors, and thickeners. This poses a serious threat to children's health (Pries et al. 2024). About 58% of staple foods in mainstream US supermarkets are ultra‐processed, containing over 41% ultra‐processed food markers compared to European foods. This underscores the high consumption of processed foods and artificial flavors in the United States (Amaraggi et al. 2024). Increased consumption of ultra‐processed foods, which are low in nutrients and high in unhealthy additives, can lead to obesity and chronic diseases. Additionally, environmental concerns arise from the use of added flavors that increase overeating and hamper flavor–nutrient learning (Neumann and Fasshauer 2022). Higher consumption of artificial sweeteners, particularly aspartame and acesulfame‐K, is related to an increased risk of cardiovascular and cerebrovascular diseases (Debras, Chazelas, Sellem, et al. 2022). Additionally, artificial sweeteners intake, especially saccharin and acesulfame‐potassium, has been involved in increasing the risk of Type II diabetes by altering the gut microbiome (Li 2023). These sweeteners also contribute to the horizontal gene transfer of antibiotic resistance genes, posing further health risks as their usage increases (Yu et al. 2022). Additionally, it is important to recognize that some natural flavor compounds formed during high‐temperature processing, such as acrylamide in baked or fried starchy foods and heterocyclic amines (HCAs) in grilled meats, may also pose potential health risks (Vignesh et al. 2024). While these compounds contribute to the characteristic flavors of certain foods, they have been associated with adverse effects such as inflammation, cancer risk, and metabolic disorders, underscoring the need for a more nuanced evaluation of both artificial and naturally occurring flavor substances (Edna Hee et al. 2024).

### 6.2 Barriers to Adopting Flavor‐Focused Nutritional Interventions

Several obstacles hinder the adoption of flavor‐focused nutritional interventions. Economic constraints make healthier, natural, and flavorful foods more expensive and less accessible, particularly for some individuals and communities (Zenk et al. 2022). A report by Goodman (2017) highlighted that limited awareness and education about the benefits of natural flavors impede these efforts, as many people may not fully understand their health advantages (Goodman 2017). Accessibility is another issue; fresh and natural ingredients may not be readily available or easily accessible in all areas, making it difficult for people to incorporate them into their diets (Zenk et al. 2022). Moreover, the strong marketing of processed foods with artificial flavors often overshadows efforts to educate the people on healthy eating. This is particularly concerning for young children, as studies have shown that most educational websites used for remote learning feature advertisements for unhealthy foods, giving these products unprecedented access to young audiences (Emond et al. 2021). There are perceived taste differences; people accustomed to excessively processed foods may find it challenging to accept the subtle and less concentrated taste flavors of foods, as shown in a study on artificial flavor preference over natural flavors of vanilla in soft ice cream (Khantyanissa and Ervina 2024). The increasing use of natural extracts rich in nitrate and nitrite as co‐products for replacing artificial additives in meat products has raised concerns about their effects on color, lipid oxidation, and sensory properties (Sichetti Munekata et al. 2024). Recent research highlights significant gaps in knowledge regarding flavor mechanisms and their health implications. Although the use of flavor molecules in the food industry is common, research in this area has been limited due to a lack of comprehensive databases and standard descriptions (Kou et al. 2023). Large gaps remain in understanding the health effects of artificial versus natural flavors, flavor‐gut microbiome interactions, and the effects of flavors on eating behaviors and diseases.

## 7 Innovative Approaches and Future Directions

### 7.1 Recent Advancements in Food Processing Technology

Recent developments in food processing have ensured that natural flavor enhancers emerge with positive health and nutritional effects. This follows breakthroughs such as fermentation and enzyme hydrolysis that are driving the synthesis of umami compounds which make food products more appetizing while potentially reducing the need for synthetic flavor enhancers (Ruan et al. 2022). Emerging food processing technologies, such as high‐pressure processing and ultrasound processing, have the potential to greatly improve food quality, maintain nutritional components, and extend shelf‐life without affecting sensory features (Sharma et al. 2024). Some of the emerging approaches for developing texture‐modified foods for elderly individuals with dysphagia include nonthermal technologies, gelation, and 3D printing, with a focus on flavor enhancement and nutritional enrichment (Liu et al. 2024). Flavors, which are natural products, contain bioactive compounds that act as natural antioxidants, anti‐inflammatory, and antimicrobial agents. These include vanilla, coffee, cardamom, saffron, cinnamon, and others, which may be useful in preventing diseases like cancer and neurodegenerative disorders (Dibwe et al. 2023; Guo et al. 2024; Hossain et al. 2025; Tušek et al. 2024). These compounds may also improve the shelf life of food when used as additives. With the growing interest in natural flavors, research into their functional properties is expanding with potential applications in nutraceuticals and functional foods (An et al. 2022; Gupta et al. 2023). Table 5 provides a detailed overview of recent advancements and potential interventions in food processing, emphasizing the significant developments that are emerging within this evolving field.

**TABLE 5:** Recent advancements and potential interventions in food processing.

Advancement/intervention	Description	Potential benefits for well‐being	Examples of application	Reference
Flavor modulation techniques	Methods to alter or enhance flavors in foods using natural or artificial means	Improved taste, reduced need for unhealthy additives, increased acceptance of healthy foods	Use of flavor enhancers in low‐sugar beverages	(McBey and Nadathur 2024)
Umami flavor enhancement	Incorporation of natural umami compounds to enhance savory taste without increasing sodium content	Reduces sodium intake, enhances palatability, and increases appetite in individuals with taste impairments	Use of mushrooms, tomatoes, and seaweed extracts in low‐sodium foods	(Hossain et al. 2024)
Natural flavor enhancers	Compounds derived from natural sources to enhance food flavors	Improved taste perception without added sugars or salts	Use in low‐sugar beverages, reduced‐sodium snacks	(Cordero‐Soto et al. 2024; Vasilaki et al. 2022)
Flavor release kinetics	Studying the release rates of flavors during consumption	Optimized flavor delivery, improved sensory experience	Chewing gum, flavored beverages	(Jia et al. 2021; Meyers 2022)
Plant‐based flavor innovations	Developing flavors that mimic meat and dairy products using plant‐based ingredients.	Support for vegetarian and vegan diets, reduced environmental impact	Plant‐based meats, dairy‐free cheeses	(Variyar and Mishra 2024)
Functional flavors	Creating flavors with added health benefits, such as probiotics or antioxidants	Enhanced nutritional value, support for digestive health and immunity	Functional beverages, fortified snacks	(An et al. 2022; Gupta et al. 2023)
Flavor pairing algorithms	Using data science to identify complementary flavor combinations	Enhanced culinary experiences, innovative food products	Recipe development, gourmet food pairing	(Zhou et al. 2024)
Flavor encapsulation	Encapsulating flavors in protective coatings to enhance stability and controlled release	Improved flavor retention, prolonged shelf life, and targeted delivery in the digestive system	Functional foods, dietary supplements, and pharmaceuticals	(English et al. 2023)
Biotechnology in flavor production	Utilizing microbial fermentation and genetic engineering to produce natural flavors	Sustainable and scalable production of flavors, reduced environmental impact	Plant‐based foods, eco‐friendly flavor production	(Abd‐Alrahman et al. 2024)
Ai‐driven flavor development	Exploring machine learning algorithms to analyze vast datasets of flavor compounds and consumer preferences	Personalized flavor experiences, innovative and unique food products	Custom beverage formulations, AI‐generated recipes	(Thakur and Sharma 2024)
Flavor enhancement through fermentation	Applying traditional and modern fermentation techniques to develop rich and complex flavors	Improved gut health, enhanced nutritional value	Fermented beverages, probiotic‐rich foods	(Hernández‐Velázquez et al. 2024)

### 7.2 Potential Interventions for Enhancing Well‐Being Through Flavor

Flavor may be used for the promotion of well‐being through new strategies. Among the most promising approaches is the application of natural flavoring agents. Natural agents represent bioactive compounds with health‐promoting properties (Badiche‐El Hilali et al. 2024; Gan et al. 2023), and by incorporating such flavors into foods, we can potentially improve dietary habits and overall health outcomes. This strategy includes developing appealing flavor profiles to improve dietary adherence and formulating functional foods and nutraceuticals designed to support specific health outcomes (Gupta et al. 2023). One effective intervention could be flavored oral nutritional supplements to improve the nutritional intake of elderly patients, or flavor masking strategies to make healthier food more palatable (Gallego et al. 2022; Liu et al. 2024). Moreover, ongoing research in flavor technology aims to develop products that align more closely with individual taste preferences and dietary needs, making it a promising avenue for health promotion (Aghababaei and Hadidi 2023; Tepper and Barbarossa 2020; Tušek et al. 2024). Studies have shown that the use of herbs and spices to enhance flavors in healthier foods can help consumers reduce their intake of saturated fat and sodium while maintaining acceptable taste (Petersen et al. 2024). The post‐COVID‐19 world has sparked innovative approaches to flavor and food development, with botanical and floral flavors becoming highly preferred for their perceived benefits to consumer well‐being (Ayseli 2023). Future research in this sector will likely focus on natural flavors with health benefits, using functional foods with bioactive compounds to individualize flavor experiences. This will connect the fields of food science, nutrition, psychology, and genetics.

## 8 Global Food Flavor Market: Current Overview

The food flavor market is growing fast, driven by increasing consumer demand for natural, healthy, and functional flavors (Gan et al. 2023). To meet these demands, the industry is actively innovating, creating new flavors, and improving existing ones. Technologies like encapsulation are being utilized to protect flavors from deterioration during processing and storage (Anil et al. 2023). Recent reports indicate that the market is characterized by diverse flavors and applications, showing the increasing consumer demand for innovation and variety within the food and beverage industry (Gan et al. 2023). One significant trend is the growing preference for natural and organic flavors, particularly among health‐conscious consumers who are looking for products with fewer synthetic additives (Kalyani and Prabhavathi 2023). This has led to enhanced use of natural flavor extracts obtained from fruits, herbs, and spices (Tamilarasi and Krishnakumar 2023). Improvements in flavor technology, such as flavor encapsulation and extraction methods, have introduced numerous possibilities for the development of new flavors (Dong et al. 2023; Gan et al. 2023). Flavors play a significant role in boosting the sales of food products by enhancing taste appeal, differentiating products, and creating brand loyalty. Unique and pleasant flavors attract consumer attention and drive repeat purchases. These innovative flavors, being either seasonal or limited edition, will generate buzz and sell (Günden et al. 2024). Flavors also contribute to emotional bonding between consumers and products by evoking positive emotions and memories, which in turn drive sales and contribute to a brand's success (De Pelsmaeker et al. 2022). The global food flavor market is expected to continue growth because of ongoing innovation, expanding applications, and a trend toward natural ingredients. Companies will focus on developing new flavor profiles and innovative flavor technologies that align with changing consumer tastes. The dynamic and competitive nature of the market offers numerous opportunities for growth and innovation.

## 9 Conclusion

Flavor perception is influenced by genetic factors, age, regional background, cultural context, and gender, all of which significantly impact dietary choices and overall health. It affects physiological and emotional reactions, with complex brain mechanisms guiding food choice. Acknowledging the role of these factors places natural flavors in the spotlight, as they have the ability to increase the adoption of healthier foods and reduce dependency on artificial additives that can have negative impacts. Greater demand for natural and organic flavor by health‐conscious consumers is a key trend, despite setbacks in terms of low bioavailability and lack of sufficient clinical trials to confirm their efficacy. Nevertheless, new advances in food processing technology, including microencapsulation and better extraction methods, are promising means of improving flavor stability and sensory quality. These advances make healthier products more palatable and acceptable. The use of flavor in food reformulation, nutrition intervention, and nutrition education plays a key role in promoting healthier and more sustainable eating. Subsequent research must target human trials for optimizing flavor delivery and dosage techniques, and industry‐specific guidelines for implementing these findings. Understanding how genetic traits (such as TAS2R38 and TAS1R2) interact with the environment will guide customized flavor experience development. Through the integration of food science, nutrition, psychology, and genetics, the field can make significant progress in public health and dietary choice among diverse populations. Moreover, governments worldwide should enforce stricter regulations on the use of artificial additives, ensuring that industry standards are adhered to and that these additives do not exceed safe limits, thereby safeguarding consumer health.

## Author Contributions

**Md. Sakhawot Hossain:** conceptualization (equal), data curation (lead), investigation (lead), methodology (lead), resources (lead), validation (lead), visualization (lead), writing – original draft (lead). **Md Abdul Wazed:** conceptualization (equal), methodology (equal), validation (equal), visualization (equal), writing – review and editing (equal). **Sharmin Asha:** resources (equal), visualization (equal), writing – review and editing (equal). **Md Alomgir Hossen:** resources (equal), writing – review and editing (equal). **Sk. Nur Muhammad Fime:** visualization (supporting), writing – review and editing (equal). **Shamiha Tabassum Teeya:** visualization (supporting), writing – review and editing (equal). **Lubna Yeasmin Jenny:** writing – review and editing (equal). **Diptho Dash:** visualization (supporting), writing – review and editing (equal). **Islam Md Shimul:** conceptualization (equal), funding acquisition (lead), supervision (lead), validation (equal), writing – review and editing (equal).

## Ethics Statement

The authors have nothing to report.

## Consent

The authors have nothing to report.

## Conflicts of Interest

The authors declare no conflicts of interest.

## Data Availability

No new data were generated or analyzed in this review. Data supporting the findings of this study are available in the referenced works.
